# Potential role of strike-slip faults in opening up the South China Sea

**DOI:** 10.1093/nsr/nwz119

**Published:** 2019-08-20

**Authors:** Chi-Yue Huang, Pinxian Wang, Mengming Yu, Chen-Feng You, Char-Shine Liu, Xixi Zhao, Lei Shao, Guangfa Zhong, Graciano P Yumul

**Affiliations:** 1 State Key Laboratory of Marine Geology, School of Ocean and Earth Science, Tongji University, Shanghai 200092; 2 Department of Earth Science, Cheng Kung University, Tainan 701; 3 Institute of Oceanography, Taiwan University, Taipei 10617; 4 Earth and Planetary Sciences, University of California, Santa Cruz, CA 95064; 5 School of Environmental Science and Management, University of the Philippines Los Banos College, Los Baños Laguna, Philippines 4031

**Keywords:** South China Sea, Huatung Basin, Eurasian continent, strike-slip fault, plate boundary

## Abstract

Radiometric dates of key rock units indicate that a remnant Late Mesozoic ocean of the Huatung Basin is still preserved today east of the South China Sea (SCS). We integrate regional geology with a Cretaceous oceanic basement in the vicinity of the Huatung Basin to reconstruct the Huatung Plate east of the Eurasian continent. Results of geophysical investigations, four expeditions of deep-sea drilling and a renaissance of regional geology allow us to propose a hypothesis that the mechanism responsible for the SCS opening was raised from strike-slip fault on the east. The hypothesis suggests that the SCS opening could highly relate to the strike-slip faults inherited from Late Mesozoic structures onshore–offshore the SE Cathaysia Block to develop rhombic-shaped extensional basins *en echelon* on the thinned Eurasian continental crust in the Early Cenozoic. It was followed by sinistral strike-slip movements along the boundary between the Eurasian Plate and the Huatung Plate driven by oblique subduction of the Huatung Plate to the northwest coupled with slab-pull force by southward subduction of the Proto-SCS to open up the triangle-shaped oceanic East Sub-basin in the Early Oligocene (33/34 Ma). The spreading ridge then propagated southwestward in the step-over segment between the Zhongnan-Lile and the Red River strike-slip fault systems to open the triangle-shaped oceanic Southwest Sub-basin by 23 Ma. The plate boundary fault was subsequently converted into the Manila Trench when the Eocene Sierra Madre arc of the Huatung Plate had moved from the south to its present latitude by the Middle Miocene.

## INTRODUCTION

**Figure 1:**
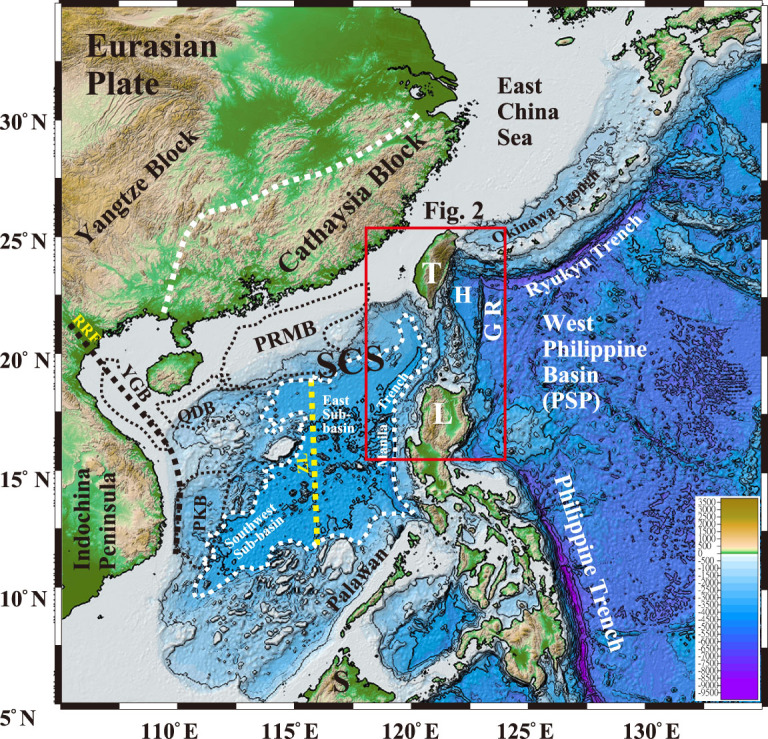
Locations of the South China Sea and adjacent regions along the Eurasian Plate edge. GR, Gagua Ridge; H, Huatung Basin; L, Luzon; PRMB, Pearl River Mouth Basin; QDB, Qiongdongnan Basin; RRF, Red River–East Vietnam fault system; PSP, Philippine Sea Plate; YGB, Yinggehai Basin; PKB, Zhongjiannan Basin; ZL, Zhongnan-Lile strike-slip fault.

The South China Sea (SCS) is the largest marginal sea opened along the Eurasian Plate edge (Fig. 1). The SCS has long been proposed as a mini non-volcanic Atlantic-type passive continental margin [1] which is commonly associated with an exposure of serpentinite exhumed from the upper mantle in the Iberian-type low-spreading non-volcanic margin. Recent IODP expeditions conducted in the SCS (Expeditions 349/367/368/368X), however, demonstrated a rapid transition from the continental breakup to the formation of an igneous oceanic crust without serpentinite exhumation on the ocean side of the continent–ocean transition zone [2]. This would suggest that the mechanism to open up the SCS along the Eurasian continental plate edge differs significantly from the way to open an ocean such as the Atlantic from a supercontinent [3,4]. The drilling results are thus at odds with the existing mechanisms proposed previously to open up the SCS. This demands reconsidering how the SCS was opened based on new results obtained from IODP expeditions.

In this paper, we integrate regional geology and new age dates on key geological units east of the SCS and use the results to suggest a new hypothesis for the opening mechanism of the SCS. The new hypothesis suggests that the major mechanism to open up the SCS could be inherited from the Late Mesozoic strike-slip structures onshore–offshore at the SE Cathaysia Block along the Eurasian continental plate edge and lithospheric stretching along the plate boundary between the Eurasian Plate and the Huatung Plate coupled with slab-pull force by the southward subduction of the Proto-SCS in the Eocene–Oligocene.

### Fundamental geology features of the SCS

Intensive marine surveys demonstrated that the geology of the SCS is characterized by: (i) the occurrence of Eocene rhombic-shaped extensional basins *en echelon* on the Mesozoic magmatic basement with a thinned continental crust (30–20 km) in shelf-slope regions [5,6], (ii) the largest triangle-shaped marginal sea with a 5- to 6-km-thick oceanic crust of the Oligocene~Mid-Miocene age along the Eurasian Plate edge [7,8], (iii) being divided into two oceanic sub-basins by a sinistral N–S-trending Zhongnan-Lile strike-slip fault in the middle part [9] (Fig. 1) and (iv) a mid-ocean ridge spreading first in the East Sub-basin (33/34–15 Ma) then propagating landward to the taper side of the Southwest Sub-basin (23–16 Ma) [7,8]. Along the Asian continental margin, the SCS is also featured by (v) no convergent structure in its ocean side when it was opening in the Early Oligocene and (vi) having experienced tectonics from rifting through spreading to subduction in its short-lived basin history.

Basin geometry and the direction of ridge propagation are two of the fundamental geology features for evaluating the driving mechanism responsible for opening up the SCS. The basin geometry represents the kinematic results of force; it will thus provide the key to understanding the mechanism and how it operated to develop the SCS, whereas ridge propagation will provide additional evidence for checking whether or not the proposed mechanism to develop the SCS is feasible.

### Opening mechanisms proposed previously

In the past three decades, three main mechanisms have been proposed for the formation of the SCS: (i) extrusion of the Indochina Block along the Red River–East Vietnam fault (RRF in Fig. 1) between the Indochina Block and the Yangtze Block in the west [1,7], (ii) slab pull by subduction of the Proto-SCS in the south [10,11] and (iii) upwelling of the deep-mantle plume within the SCS [12,13].

These main mechanisms proposed previously are helpful for understanding the orientation and sequence of the magnetic anomaly lineations of the SCS, but did not interpret successfully why the SCS was opened as a triangle-shaped oceanic basin with a wide open side in the east before opening the Southwest Sub-basin in the narrow taper side in the southwest. For example, if the RRF system (Fig. 1) played the major role in the SCS opening, one would expect that (i) the opening of the oceanic Southwest Sub-basin (23–15 Ma) in the west would be earlier, because it was much closer to the RRF than the East Sub-basin (33/34–15 Ma) that was far away from the RRF (Fig. 1); (ii) the long axis of the SCS basin would be orientated in the NW–SE to N–S direction parallel~subparallel to the Yinggehai Basin–Zhongjiannan Basin (YGB and PKB in Fig. 1) axes developing within the step-over segments of the RRF strike-slip fault system, instead of the E–W direction subparallel to the Pearl River Mouth Basin (PRMB in Fig. 1) in the northern shelf-slope region; and (iii) the spreading ridge of the SCS oceanic basin would propagate northeastward instead of southwestward. All these contradictions indicate that the extrusion model along the intra-continental RRF system could play only a minor role rather than a major one for opening up the SCS.

Southward subduction of the Proto-SCS in the Early Oligocene resulted in the formation of the Cagayan volcanic ridge on the Borneo micro-continental plate, which was later counter-clockwise rotated to collide with the Central Philippines to the east in the late Oligocene–Early Miocene and also a collision with the North Palawan Continental Terrane to the north in the Early Miocene [10,11]. The southward subduction of the Proto-SCS would certainly contribute to a tectonic slab pull for opening of the SCS north of the Palawan Continental Terrane in the Early Oligocene. However, the collisions in the east and the north would decelerate the southward subduction of the Proto-SCS and thus there would be no active function of the slab-pull effect to open the SCS since the Early Miocene. But the SCS opened continuously from the Early Oligocene to the Middle Miocene. Consequently, southward subduction of the Proto-SCS could not be a major mechanism alone to open up the SCS. There would need another main mechanism to drive the lithospheric stretching of the SCS.

**Figure 2:**
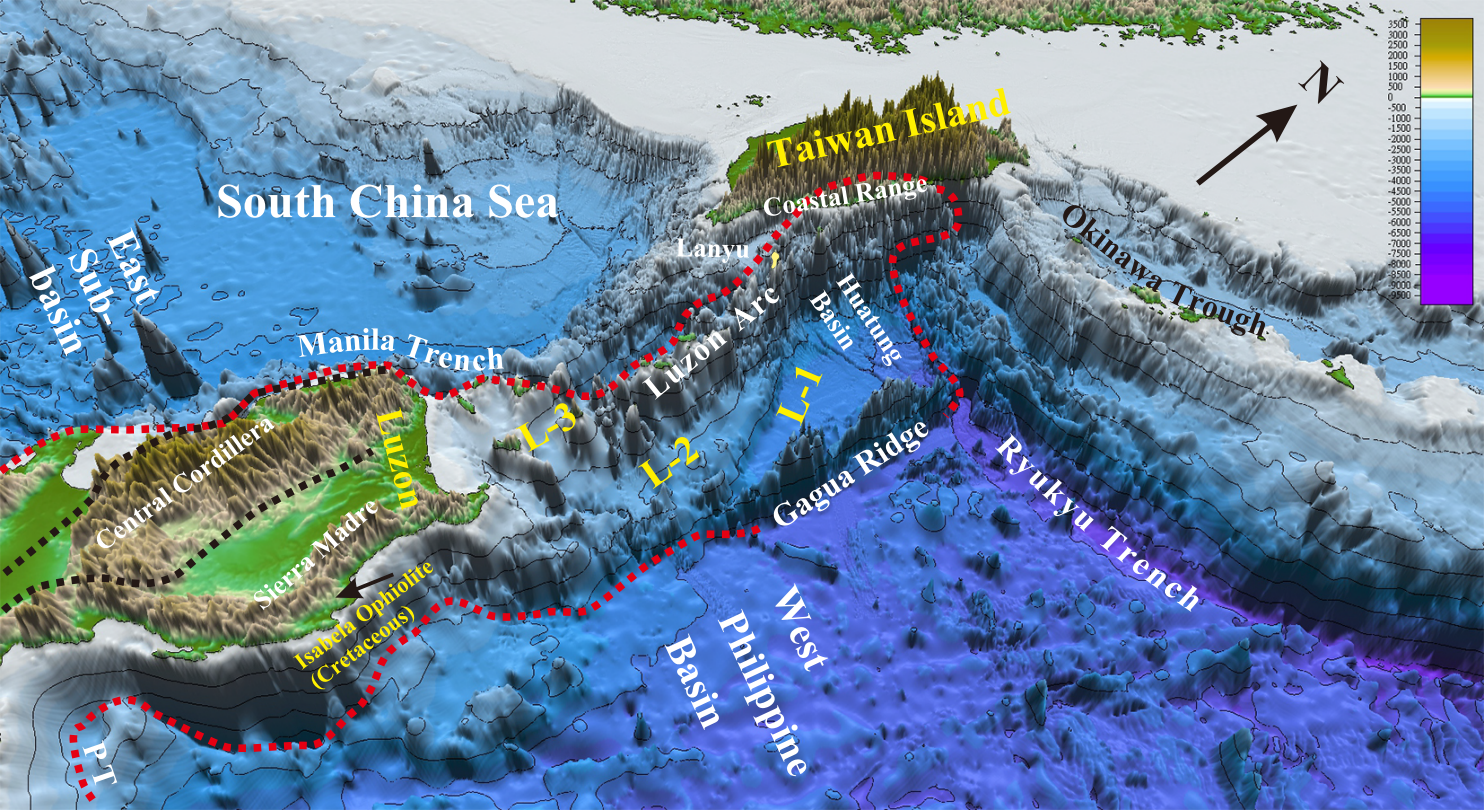
Configuration of the Huatung Plate (area marked by red rots) between the South China Sea and the West Philippine Basin showing also distributions of three morpho-tectonic units (L-1, L-2 and L-3) offshore between Taiwan and Luzon and three ophiolite belts onshore at the northern Luzon Island [17]. Location of Fig. 2 is shown in Fig. 1.

**Figure 3:**
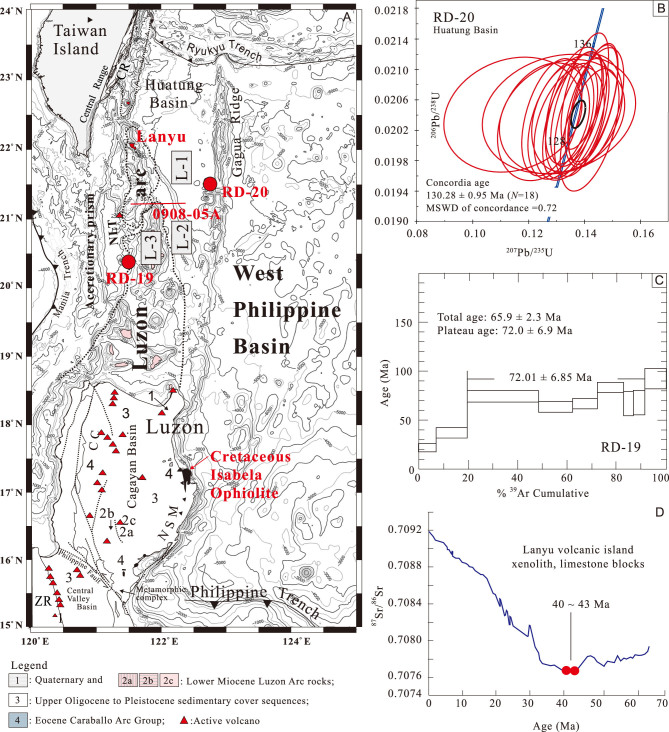
(A) Simplified geological maps of Luzon–SE Taiwan locations of two studied Cretaceous rock samples (red dots) dredged by *R/V Vema* during Cruise 3609 in 1980 from the Huatung Basin (RD-20) and the basement beneath the Luzon arc (RD-19); (B) age dating on rock Sample RD-20 determined by zircon grains (130 ± 0.95 Ma) separated from the gabbro using the SIMS method; (C) ^40^Ar/^39^Ar age of multiple grains of amphibole in gabbro (plateau age: 72.0 ± 6.9 Ma) of rock Sample RD-19; (D) ^87^Sr/^86^Sr isotopic age of two xenolith limestone blocks scattered in the Lanyu volcanic island of the Luzon arc. CC, Central Cordillera; NSM, Northern Sierra Madre; ZR, Zambales Range. L-1, L-2 and L-3 are three tectono-morphological units offshore between Taiwan and Luzon.

On the other hand, various age-reliable records of seamount eruption events presumably due to the mantle pluming or upwelling [14] in the SCS were not earlier than the earliest eruption event of the mid-ocean ridge basalt in the SCS [15]. This suggests that, if the mantle plume or upwelling was one of the important mechanisms to open up the SCS, more reliable age dates on seamount basalts are needed to prove that these seamount eruptions occurred earlier than the SCS seafloor spreading at 33/34 Ma.

Seismic surveys and fault-stress measurements showed that developments of the great PRMB in the northern shelf-slope of the SCS were all highly related to the NE–SW to ENE–WSW trending strike-slip fault systems inherited from the Mesozoic structures onshore–offshore the Cathaysia Block along the Eurasian Plate edge [6,16]. The mechanism models proposed previously overlooked any possible mechanism that might originate from the east because the configuration of the eastern SCS has been consumed by the plate convergence along the Manila Trench since the Mid-Miocene. The key to understanding the mechanism of the SCS opening relies upon an integration of regional geology and age dating on key geological units east of the SCS, especially the geological nature of the Huatung Basin and the succession beneath the Mid-Miocene~Pleistocene Luzon arc offshore between Taiwan and Luzon.

## RESULTS

### New age dating on dredged rocks from the Huatung Basin (Sample RD-20)

 The Huatung Basin (18°N−23°N, 122°E−123°E, Fig. 1) is a parallelogram-shaped small ocean basin (22,300 km^2^; water depth: −4000 ~ −5000 m; 5- to 7-km-thick oceanic crust [17]) between the SCS and the West Philippine Basin (Figs 1 and 2). It is bounded by the Luzon arc in the west and the Gagua Ridge in the east, and connects southward with the Luzon Island of the Philippine Mobile Belt (Fig. 2). At present, the Huatung Basin, Gagua Ridge and West Philippine Basin are all subducting northward beneath the rifting Eurasian Plate along the Ryukyu Trench (Fig. 2).

The crust age of the Huatung Basin was first proposed to be the Eocene as part of the West Philippine Basin by interpretation of paleomagnetic lineations [18]. However, ^40^Ar-^39^Ar dating on a single grain of amphibole in a gabbro block dredged by *R/V Vema* in 1980 showed that the crust of the Huatung Basin was of an Early Cretaceous age (131–119 Ma; Fig. 3A) [19]. This radiometric Early Cretaceous age is supported by our new independent dating results on 18 zircon grains (concordant age of 130.28 ± 0.95 Ma; Fig. 3B and Supplementary Table 1) from the same dredged sample determined using a SIMS machine.

**Figure 4:**
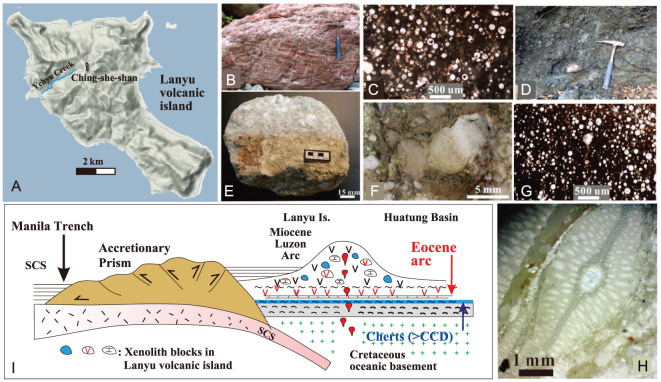
(A) Topography of the Lanyu volcanic island and location of the Yehyu Creek; (B) xenolith blocks of a deep-sea ribbon cherty block; (C) the Early Cretaceous (115 Ma) radiolarian microfossils in a cherty block [20]; (D) field occurrence of xenolith ribbon cherty blocks enclosed by volcanic agglomerates; (E) and (F) xenolith Eocene deep-sea limestone block with (G) white spots of radiolarian cysts; (H) larger foraminifera *Lepidocyclina* in a young-aged limestone block; (I) interpretation for the occurrence of various xenolith blocks in the Lanyu volcanic island of the Luzon arc off SE Taiwan Island.

### Age dating on dredged rocks from the basement beneath the Miocene Luzon arc (Sample RD-19)

Based on bathymetric survey, the rock Sample RD-19 (20.40°N, 121.47°E; dredged by *R/V Vema* in 1980; Fig. 3A) could be collected from a steep scarp along the western flank of the Luzon arc. We re-measured the age of this dredged rock sample on multiple grains of amphiboles in gabbro using the ^40^Ar/^39^Ar dating method. The result confirms a Late Cretaceous age (plateau age: 72.0 ± 6.9 Ma; Fig. 3C and Supplementary Table 2), which is younger than what had reported previously (plateau age: 116.2 ± 4.2 and 121.2 ± 4.6 Ma) [19]. This may suggest that the hydrothermal-altered Cretaceous basement could have experienced multiple thermal events, as indicated by the sequence that the study materials were overlain by the Miocene~Pleistocene Luzon arc offshore north of the Luzon Island (Fig. 3A).

### Old xenolith blocks in the young Lanyu volcanic island of the Luzon arc off SE Taiwan

Eastward subduction of the SCS since the Mid-Miocene resulted in eruptions of the Luzon arc. The Luzon arc between Taiwan and Luzon occurs as a chain of cone-shaped volcanoes or Plio-Pleistocene small flat volcanic islands without significant deformation in a triangle-shaped region (<−4000 m) getting narrow northward offshore SE Taiwan (Fig. 2). The Lanyu volcanic island at the northern tip of the Luzon arc off SE Taiwan is composed of the Mid-Miocene~Pleistocene bedded andesitic agglomerates with intrusives and is fringed by the Latest Pleistocene-Recent reef limestone along the coast. However, there are sheared, well-diagenetic, fine-laminated to thick-bedded, large ribbon cherty blocks (3 × 5 × 3 m) scattered along the Yehyu Creek in the western side of the island (Fig. 4A and B). The cherty blocks contained the Early Cretaceous radiolarians cysts of the Barremian age (115 Ma; Fig. 4C) [20]. Our field survey showed that these radiolarian-bearing cherts occurred as xenolith blocks enclosed by volcanic agglomerates with hydrothermal fault gauges (Fig. 4D) presumably near the crater.

In addition, there are also old- and young-aged limestone blocks scattered along the creek. The young-aged shallow-marine limestone blocks contained larger foraminifers *Lepidocyclina* similar to the Kangkou Limestone (~5.2 Ma) in the Coastal Range, eastern Taiwan (Figs. 2 and4H) [21]. The old-aged deep-marine limestone blocks with radiolarian fossils had never been found in the Luzon arc before. For a precise age determination of these old-aged deap-sea limestone blocks, we measured their Sr-isotope values using the TIMS technique. The ^87^Sr/^86^Sr values are 0.707621 and 0.707639, respectively. Comparing to the global seawater Sr-isotopic curve deduced from planktonic foraminiferal shells [22], the results show an Eocene age (40–43 Ma; Fig. 3D). The Eocene deep-sea chert with radiolarian fossils and the deep-sea limestone with chert beds were found in the Caraballo Formation (volcanic arc) overlying the Early Cretaceous ophiolite in the Sierra Madre, eastern Luzon [23,24], and the Akistero Formation in the northern Luzon Island [25] below the Luzon arc-related rock sequences, respectively. These results confirm that there was an Early Cretaceous oceanic basement unconformably overlain by Eocene deep-sea carbonates beneath the Miocene Luzon arc (Fig. 4I). The xenolith blocks were brought to the Lanyu volcanic island surface by the late Luzon arc volcanism (Fig. 4I).

**Figure 5:**
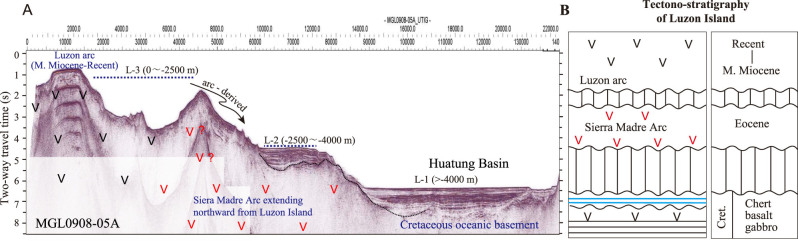
Interpretation of (A) a W–E seismic profile (MGL 0908-05A) across the Luzon arc–Huatung Basin showing three tectono-morphological units (L-1, L-2 and L-3) offshore that correspond to (B) three tectono-stratigraphy exposed onshore the north Luzon Island. Location of the seismic profile is shown in Fig. 3.

## DISCUSSION

### Late Mesozoic oceanic basement exposed in the Luzon Island

The Luzon Island of the Philippine Mobile Belt is composed of five morpho-tectonic units (Fig. 2) [26]. Among them, the Zambales Range and the Central Valley Basin (Fig. 3A) could be moved from the south along the Philippine fault. Tectonic setting of these two units in the western Luzon Island could relate to eastward subduction/collision of the Proto-SCS [26], whereas the other three morpho-tectonic units in the eastern Luzon Island have a similar stratigraphy which can be grouped into three major tectono-stratigraphic units unconformably in ascending order: the Late Mesozoic ophiolitic basement with overlying deep-sea cherts (130–110 Ma) [24] in the lower part, the Eocene-Early Oligocene volcanics/intrusives in the middle part and the Middle Miocene-Recent volcanics in the upper section (Fig. 5) [23]. A complete oceanic upper-mantle–crust sequence of the Isabela ophiolite (Fig. 2), for example, represents the basement of the Northern Sierra Madre, whereas the Dos Hermanos Mélange with dismembered ophiolite sequences marks the basement of the Luzon Central Cordillera (Fig. 2). The chert beds overlying the Isabela ophiolite or within the Dos Hermanos Mélange contained the Cretaceous radiolarian microfossils [23,24]. These Late Mesozoic subduction-related SSZ-ophiolite basements represented arc–forearc–backarc slices above a westward-subducting Mesozoic ocean to the east [23]. However, the present ocean east of the Philippine Mobile Belt is the Eocene West Philippine Basin (Fig. 1). This suggests that the Mesozoic ocean east of the Philippine Mobile Belt had been consumed entirely by the Late Cenozoic. Consequently, the origin of the Philippine Mobile Belt was considered ambiguously as the Proto-Philippine Sea Plate [26].

The Cretaceous ophiolitic basement and the overlying deep-sea chert sequence in northern Luzon are comparable to the Cretaceous oceanic basement either of the Huatung Basin (Sample RD-20) or beneath the Luzon arc (Sample RD-19) and the Early Cretaceous xenolith cherty blocks in the Lanyu volcanic island. This suggests a comparable stratigraphy between onshore and offshore the Luzon Island, and a remnant part of the great Mesozoic ocean is still preserved today east of the SCS [19].

### The Eocene Sierra Madre arc unconformably underlying the Mid-Miocene Luzon arc

Regional geology study pointed out that the Luzon Central Cordillera and the Northern Sierra Madre were combined together as a single Eocene magmatic arc (= the Sierra Madre arc) before backarc spreading to generate the inter-arc Cagayan Basin in the Late Oligocene–Early Miocene [27]. Different stratigraphic names were applied to the thick (up to 6000–10 000 m [28]) and widely exposed Eocene arc sequences (= Caraballo Arc Group) in the northern Luzon Island (Fig. 3A). Paleomagnetic measurements further revealed that the Eocene Sierra Madre arc remained in a low latitude near the Equator in the Eocene and it was then moved rapidly northward to the present latitude in the last 20–25 Ma [23]. The plate-motion record of the Luzon Island could be highly contrasted with the records of the Philippine Sea Plate [29] if they were different plates.

The flat topography and low elevation of the Cagayan Valley Basin and the Central Valley Basin indicate no intensive deformation in the northern Luzon Island post the Oligocene except strike-slip faulting along the Philippine fault since the late Miocene. Therefore, geology of the Luzon Island (13°N−18°N) should extend northward offshore. Bathymetry mapping and seismic surveys in the region between Taiwan and Luzon (18^o^N−22^o^N) show the occurrence of three tectono-morphological units across the section from the Luzon arc eastward to the Huatung Basin: upper unit (water depth: 0 ~ −2500 m), middle unit (water depth: −2500 ~ −4000 m) and lower until (water depth: > − 4000 m; Figs 2, 3 and 5). Due to lack of compressive deformation, these three tectono-morphological units offshore (L-3, L-2, L-1 in Fig. 2) could correspond to three main tectono-stratigraphic units stacked upward onshore of the northern Luzon Island, respectively (Fig. 5). We regard that the uppermost tectono-morphological unit (L-3) is obviously equivalent to the youngest tectono-stratigraphic unit of the Mid-Miocene~Pleistocene Luzon arc. The middle tectono-morphological unit (L-2) could correspond to the Sierra Madre arc composed mainly of the Eocene Caraballo Arc Group. This Eocene Sierra Madre arc represented a fossil convergent zone by a westward subduction of a consumed Mesozoic ocean east of the Philippine Mobile Belt [27]. The lowest tectono-morphology unit (L-1, the Early Cretaceous oceanic crust of the Huatung Basin) is equivalent to the Early Cretaceous ophiolitic basement with radiolarian cherts in the north Luzon Island (Fig. 5).

### Reconstruction of the Huatung Plate east of the Eurasian continent since the Early Cenozoic

**Figure 6:**
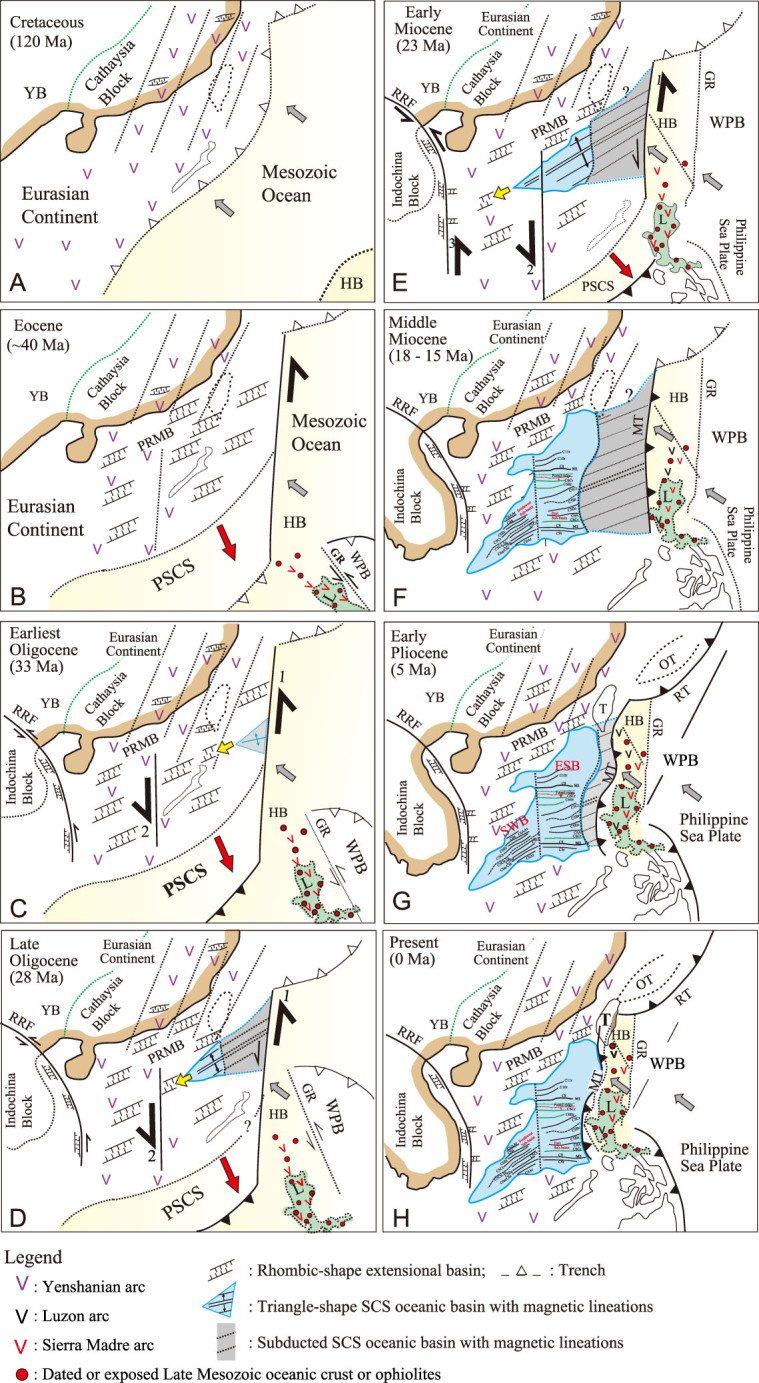
A proposed strike-slip mechanism to open up the SCS and tectonic processes (A-H) from rifting through spreading to subduction of the SCS. A great Mesozoic ocean (yellow color) was progressively consumed when the SCS (blue and gray colors) and the WPB (white region) were developing on both sides of the Huatung Basin. For details of the evolution processes, see Discussion. ESB, East Sub-basin; GR, Gagua Ridge; HB, Huatung Basin; L, Luzon; MT, Manila Trench; OT, Okinawa Trough; PRMB, Pearl River Mouth Basin; PSCS, Proto-South China Sea; RRF, Red River–East Vietnam fault; RT, Ryukyu Trench; SWB, Southwest Sub-basin; T, Taiwan; WPB, West Philippine Basin; YB, Yangtze Block. Thick half-black arrows with numbers are the major faults to open up the oceanic SCS: 1, sinistral strike-slip fault along plate boundary; 2, Zhongnan-Lile strike-slip fault; and 3, Red River–East Vietnam strike-slip fault. Gray arrows, plate motion of the Huatung Plate and the Philippine Sea Plate; red arrow, slab-pull force by southward subduction of the Proto-SCS; yellow open arrow, southwestward propagation of spreading ridge.

Although the Mesozoic ocean was generally considered to be consumed entirely by the Late Cenozoic due to subduction/collision around the Eurasian Plate edges, an Early Cretaceous oceanic basement is still preserved today between two Cenozoic marginal seas of the SCS in the west and the West Philippine Basin in the east (Figs 2 and 5). This allows us to put the regions with the Early Cretaceous oceanic basement covered by a similar stratigraphy in the vicinity of the Huatung Basin together as the Huatung Plate (Fig. 2). The Huatung Plate orientates in an N–S direction, ~300 km wide and >1000 km long, including the Huatung Basin, the Philippine Mobile Belt (at least the Luzon Island) and the offshore region between Luzon and SE Taiwan (Fig. 2). The basement represents the remnant part of a great Mesozoic ocean between the Eurasian Plate and the Pacific Plate since the Late Mesozoic (Fig. 6). Unfortunately, Late Cretaceous–Eocene geo-history of the Huatung Plate was largely unknown due to a lack of strata preserved onshore the Luzon Island and no deep-sea drilling into the Huatung Basin. However, paleo-magnetic measurements in the Luzon Island revealed that the Huatung Plate remained in the equatorial region, assuming the Huatung Plate and Luzon were together, in the Eocene time [23].

### A new mechanism hypothesis to open up the SCS by strike-slip faulting along the Eurasian Plate edge

Integrating regional geology and age dates on key geological units east of the SCS, we propose a new mechanism hypothesis to open up the SCS. The new model emphasizes that the mechanism to open up the SCS was the strike-slip faulting inherited from the Late Mesozoic–Early Cenozoic structures onshore–offshore the Cathaysia Block and the lithospheric stretching along the plate boundary between the Eurasian Plate and the Huatung Plate coupled with a slab-pull force by the southward subduction of the Proto-SCS.

The mechanism responsible for the opening of the SCS could have involved the following tectonic processes (Fig. 6):

#### Strike-slip faulting inherited from Late Mesozoic structures onshore–offshore the SE Cathaysia Block

Since the Late Jurassic~Early Cretaceous, a great Mesozoic ocean had subducted obliquely beneath the Eurasia continent to develop the Mesozoic magmatic arc onshore–offshore the Cathaysia Block and an accretionary prism in the Central Range (at least 250 km east of the present position), eastern Taiwan [30] (Fig. 6A). The subduction ended by the Late Cretaceous (~90 Ma). The Huatung Plate represented the relict of this great consuming Mesozoic ocean east of the Eurasian Plate (Fig. 6B and C). Shearing structures along strike-slip faults led to formations of the Late Mesozoic-Earliest Cenozoic basin-and-range morpho-tectonic features and extensional basins *en echelon* in the Cathaysia Block inland SE China [6,16,31,32] (Fig. 6A and B). The tectonic setting of the Cathaysia Block thus changed from an active margin in the Early Cretaceous to a passive margin in the Latest Cretaceous–Early Cenozoic. Because neither an active margin nor a mid-ocean ridge existed offshore the SE Cathaysia Block in the Early Cenozoic, the southeastern Eurasian Plate would contact the Huatung Plate along a transform fault in the Early Cenozoic [33,34] (Fig. 6B).

#### Development of rhombic-shaped extensional basins on thinned continental crust in the Paleocene–Eocene

In such a tectonic scenario, the SCS was rifted initially by extension accommodated by these NE–SW extensional faults inherited onshore and offshore the SE Cathaysia Block in the Paleocene–Eocene (Fig. 6B). The Eocene extensional basins developed *en echelon* first on the thinned Eurasian continental crust, similar to the early opening of the Japan Basin [35], by mechanisms of magmatic underplating [4] and possibly by mantle upwelling [14,34]. They were bounded by normal ENE–WSW-striking border faults offshore the SE Cathaysia Block (Fig. 6B).

#### Triangle-shaped oceanic East Sub-basin opened due to a sinistral strike-slip movement along the Eurasian/Huatung Plate boundary coupled with slab-pull force by southward subduction of the Proto-SCS in the Early Oligocene

The thinned continental crust in the lower slope was stretched continuously by the deep detachment faulting offshore the SE Cathaysia Block in the Eocene–Oligocene [2]. This gave rise to the formation of a continent–ocean transition followed by basaltic eruption south of the transition in the Early Oligocene [2]. A N–S normal seafloor spreading subsequently occurred by the lithospheric stretching along N–S-trending strike-slip fault with a sinistral sense of movement between the stable Eurasian Plate and the northwest-moving Huatung Plate driven by a northwestward oblique subduction of the Huatung Plate beneath the Eurasian Plate (Fig. 6C and D). Slab pull by the southward subduction of the Proto-SCS further triggered the oceanic opening of the East Sub-basin in the step-over segment between the Zhongnan-Lile strike-slip fault and the strike-slip fault along the Eurasian/Huatung Plate boundary in the Latest Eocene–Earliest Oligocene by 33/34 Ma (Fig. 6C and D). Southward ridge jump at magnetic lineation C6Cr (23.6 Ma) [7,8], northward plate motion and rotation of the Luzon Island [23,36] suggest a sinistral sense of relative movement between the Eurasian Plate and the Huatung Plate. Such a sinistral-sense movement between these two plates could have continued to the present day, as it was recorded by GPS measurements in eastern Taiwan and northern Luzon [37]. The basin geometry of the oceanic East Sub-basin is thus featured by a triangle-shaped sea with a wide open side facing the plate boundary fault on the east.

#### Spreading ridge propagating southwestward to open up the oceanic Southwest Sub-basin in the Early Miocene

Continuous lithospheric stretching led to the ridge propagating southwestward and the opening of the oceanic Southwest Sub-basin in the step-over segment between the RRF system and the Zhongnan-Lile strike-slip fault in the Early Miocene by 23 Ma [38] (Fig. 6E and F).

#### Eastward subduction of the oceanic East Sub-basin along the Manila Trench beneath the far-travelled Eocene Sierra Madre arc since the Mid-Miocene to become the modern configuration of the SCS

When the Eocene Sierra Madre volcanic arc of the Huatung Plate had moved from the equatorial region northward to the present latitude, the plate boundary fault was then converted to the Manila Trench in the Mid-Miocene either spontaneously due to a density contrast between the young but denser SCS oceanic lithosphere and the old but less dense Eocene Sierra Madre arc, or induced by a far-field tectonic effect along the pre-existing east-dipping structures between two plates (Fig. 6F). However, due to the eastward subduction of the SCS along the Manila Trench, the old east-dipping structures, if they did exist before the subduction, are no longer observed today [39]. As a consequence, the ~400-km-wide oceanic East Sub-basin including the early-opened part (Fig. 6C−G) presumably of the Latest Eocene age [40], had been subducted eastward along the Manila Trench [39,41] beneath the Eocene Sierra Madre arc of the Huatung Plate to become the present configuration of the SCS (Fig. 6H).

## CONCLUSION

Regional geology indicates that the Huatung Plate with the Early Cretaceous oceanic basement is still preserved presently east of the SCS. We regard that the northwestward movement of the Huatung Plate and a slab pull by the Proto-SCS southward subduction led to lithospheric stretching along the Eurasian/Huatung Plate boundary to develop the SCS. The opening processes of the SCS involved: (i) strike-slip faulting inherited from Late Mesozoic structures in the SE Cathaysia Block; (ii) development of rhombic-shaped extensional basins *en echelon* on the thinned Eurasian continental crust onshore–offshore the Cathaysia Block in the Early Cenozoic; (iii) opening the triangle-shaped oceanic East Sub-basin by lithospheric stretching and sinistral strike-slip faulting along the Eurasian/Huatung Plate boundary coupled with slab-pull force by southward subduction of the Proto-SCS in the Early Oligocene; (iv) mid-ocean ridge propagating southwestward in the Early Miocene to open the oceanic Southwest Sub-basin by the Early Miocene; and (v) eastward subduction of the oceanic East Sub-basin along the Manila Trench beneath the far-travelled Eocene Sierra Madre arc of the Huatung Plate since the Mid-Miocene to become the modern configuration of the SCS.

## METHODS

### Zircon U-Pb isotopes

Zircon grains were separated from crushed samples by using standard density and magnetic separation techniques and hand-picked carefully under a binocular microscope. Measurements of U, Th and Pb isotopes were conducted using a Cameca IMS 1280 SIMS in the Institute of Geology and Geophysics, Chinese Academy of Sciences (IGGCAS).

### ^40^Ar/^39^Ar dating

Fresh and pure feldspar and amphibolite crystals sized between 200 and 280 μm were obtained from the crushed samples by hand-picking under a binocular microscope. The separates were leached by 2 N HNO_3_ and washed using deionized water three times in an ultrasonic bath. High-resolution step-heating ^40^Ar/^39^Ar measurements were carried out using an MM5400 mass spectrometer in the ^40^Ar/^39^Ar laboratory of the IGGCAS.

### Sr-isotope measurements

A thermal ionization mass-spectrometry technique with a chemical separation method was applied for Sr-isotopic determination. The method allows the Sr size (10–20 ng) to be reduced and excellent precision (<10 ppm) to be achieved. Solutions containing >10 ng Sr were acidified, passed through an Sr^SPEC^ micro-column, 3 M HNO_3_ pre-conditioned and eluted using distilled water. We obtained an average ^87^Sr/^86^Sr = 0.710276 ± 10 ppm (2σ, *n* = 30) for SRM987 international standard.

## Supplementary Material

Supplement_DATA-2019-091_nwz119
