# Supplementary material for: Potential role of strike-slip faults in opening up the South China Sea
Source: Natl Sci Rev. 2019 Aug 20;6(5):891–901. doi: 10.1093/nsr/nwz119 (PMC8291434; doi:10.1093/nsr/nwz119)
Supplement: Supplement_DATA-2019-091_nwz119 [file supplement_data-2019-091_nwz119.docx]

Table 1. Zircon SIMS U-Pb dating of gabbro of Sample RD-20

| **Sample@** | **^207^Pb/^235^U** | **±se** | **^206^Pb/^238^U** | **±se** | **^207^Pb/^206^Pb** | **±se** | **t^207^Pb/^235^U** | **±se** | **t^206^Pb/^238^U** | **±se** | **U** | **Th** | **Th/U** | **f_206_%** |
| --- | --- | --- | --- | --- | --- | --- | --- | --- | --- | --- | --- | --- | --- | --- |
| **spot** |  | **(%)** |  | **(%)** |  | **(%)** | (Ma) | (Ma) | (Ma) | (Ma) | **(ppm)** | **(ppm)** |  |  |
| Qinghu@1 | 0.172 | 1.7 | 0.0253 | 1.6 | 0.0493 | 0.6 | 161.3 | 2.5 | 161.3 | 2.5 | 2167 | 1331 | 0.61 | 0.03 |
| Qinghu@2 | 0.167 | 1.7 | 0.0246 | 1.6 | 0.0493 | 0.5 | 156.8 | 2.4 | 156.4 | 2.4 | 1716 | 723 | 0.42 | 0.04 |
| Qinghu@3 | 0.172 | 1.8 | 0.0250 | 1.6 | 0.0500 | 0.8 | 161.3 | 2.7 | 159.0 | 2.5 | 1576 | 724 | 0.46 | 0.07 |
| Qinghu@4 | 0.170 | 1.7 | 0.0252 | 1.5 | 0.0490 | 0.8 | 159.6 | 2.6 | 160.5 | 2.4 | 2505 | 893 | 0.36 | 0.12 |
| Qinghu@5 | 0.169 | 1.7 | 0.0252 | 1.5 | 0.0488 | 0.7 | 158.9 | 2.4 | 160.4 | 2.4 | 1651 | 785 | 0.48 | 0.09 |
| Qinghu@6 | 0.173 | 1.8 | 0.0250 | 1.6 | 0.0501 | 0.8 | 161.8 | 2.7 | 159.3 | 2.5 | 1770 | 868 | 0.49 | 0.28 |
| Qinghu@7 | 0.169 | 1.6 | 0.0250 | 1.5 | 0.0491 | 0.6 | 158.9 | 2.4 | 159.2 | 2.4 | 1575 | 709 | 0.45 | 0.05 |
| Qinghu@8 | 0.174 | 2.4 | 0.0255 | 1.5 | 0.0494 | 1.8 | 162.8 | 3.6 | 162.6 | 2.4 | 2900 | 1742 | 0.60 | 0.42 |
|  |  |  |  |  |  |  |  |  |  |  |  |  |  |  |
| [RD20@01](mailto:RD20@01) | 0.132 | 3.7 | 0.0203 | 1.5 | 0.0470 | 3.3 | 125.5 | 4.3 | 129.7 | 2.0 | 140 | 75 | 0.53 | 0.63 |
| RD20@02 | 0.143 | 2.5 | 0.0206 | 1.5 | 0.0504 | 2.0 | 135.7 | 3.2 | 131.2 | 2.0 | 176 | 126 | 0.72 | 0.44 |
| RD20@03 | 0.135 | 3.8 | 0.0203 | 1.5 | 0.0481 | 3.5 | 128.2 | 4.6 | 129.5 | 1.9 | 162 | 105 | 0.65 | 0.76 |
| RD20@04 | 0.137 | 3.3 | 0.0204 | 1.6 | 0.0487 | 2.9 | 130.4 | 4.0 | 130.3 | 2.1 | 182 | 122 | 0.67 | 0.45 |
| RD20@05 | 0.140 | 2.5 | 0.0207 | 1.5 | 0.0491 | 2.0 | 133.2 | 3.1 | 132.2 | 2.0 | 259 | 142 | 0.55 | 0.21 |
| RD20@06 | 0.144 | 3.0 | 0.0205 | 1.6 | 0.0508 | 2.5 | 136.2 | 3.8 | 130.8 | 2.0 | 112 | 74 | 0.66 | 0.49 |
| RD20@07 | 0.136 | 3.0 | 0.0205 | 1.5 | 0.0481 | 2.6 | 129.6 | 3.7 | 131.0 | 2.0 | 222 | 145 | 0.65 | 0.34 |
| RD20@08 | 0.128 | 3.4 | 0.0204 | 1.5 | 0.0457 | 3.1 | 122.6 | 4.0 | 130.0 | 2.0 | 198 | 159 | 0.80 | 0.6 |
| RD20@09 | 0.114 | 8.8 | 0.0204 | 1.5 | 0.0406 | 8.7 | 109.9 | 9.3 | 130.2 | 1.9 | 150 | 101 | 0.67 | 3.35 |
| RD20@10 | 0.137 | 4.8 | 0.0204 | 1.5 | 0.0488 | 4.6 | 130.7 | 5.9 | 130.4 | 2.0 | 121 | 76 | 0.63 | 0.82 |
| RD20@11 | 0.139 | 2.9 | 0.0204 | 1.5 | 0.0495 | 2.4 | 132.2 | 3.6 | 130.0 | 2.0 | 190 | 167 | 0.88 | 0.37 |
| RD20@12 | 0.135 | 3.0 | 0.0203 | 1.5 | 0.0481 | 2.6 | 128.5 | 3.6 | 129.7 | 2.0 | 291 | 164 | 0.56 | 0.41 |
| RD20@13 | 0.125 | 5.4 | 0.0203 | 1.6 | 0.0448 | 5.1 | 119.9 | 6.1 | 129.6 | 2.0 | 105 | 64 | 0.61 | 0.86 |
| RD20@14 | 0.137 | 2.8 | 0.0205 | 1.5 | 0.0486 | 2.4 | 130.7 | 3.4 | 130.7 | 1.9 | 242 | 120 | 0.50 | 0.34 |
| RD20@15 | 0.132 | 7.0 | 0.0204 | 1.5 | 0.0470 | 6.8 | 125.9 | 8.3 | 130.0 | 1.9 | 67 | 29 | 0.44 | 1.31 |
| RD20@16 | 0.143 | 2.5 | 0.0205 | 1.6 | 0.0506 | 2.0 | 135.9 | 3.2 | 131.1 | 2.0 | 173 | 118 | 0.68 | 0.55 |
| RD20@17 | 0.131 | 8.6 | 0.0204 | 1.6 | 0.0465 | 8.4 | 124.9 | 10.1 | 130.3 | 2.0 | 46 | 20 | 0.44 | 1.07 |
| RD20@18 | 0.142 | 3.0 | 0.0202 | 1.6 | 0.0509 | 2.6 | 134.7 | 3.8 | 129.0 | 2.1 | 138 | 53 | 0.38 | 0.89 |

Table 2. Hornblende step-heating ^39^Ar/^40^Ar dating of gabbro of Sample RD-19

| Temp.  (℃) | ^40^Ar/^39^Ar | ^37^Ar/^39^Ar | ^36^Ar/^39^Ar | ^40^Ar*/^39^Ar_k_ | ^40^Ar*  (%) | ^39^Ark  (%) | Age  (Ma) | ± 2se  (Ma) |
| --- | --- | --- | --- | --- | --- | --- | --- | --- |
|  | J = 0.0040650 ± 0.0000102 | | | |  |  |  |  |
| 780 | 19.05479 | 2.90027 | 0.05524 | 2.970328 | 15.55 | 6.78 | 21.70 | 4.05 |
| 920 | 28.92223 | 29.31154 | 0.08914 | 5.045273 | 17.01 | 12.68 | 36.71 | 5.23 |
| 980^#^ | 23.49022 | 42.41952 | 0.05717 | 10.354052 | 42.49 | 28.64 | 74.55 | 5.65 |
| 1040^#^ | 22.18141 | 28.20996 | 0.05367 | 8.782122 | 38.64 | 14.01 | 63.43 | 5.58 |
| 1100^#^ | 23.68229 | 37.34820 | 0.05968 | 9.319211 | 38.10 | 9.96 | 67.24 | 5.25 |
| 1160^#^ | 28.91590 | 55.02087 | 0.07513 | 11.646294 | 38.39 | 10.65 | 83.65 | 5.15 |
| 1200^#^ | 42.73063 | 65.29632 | 0.13269 | 9.237736 | 20.42 | 4.00 | 66.66 | 12.50 |
| 1280^#^ | 62.95105 | 45.58949 | 0.19485 | 9.371827 | 14.31 | 4.49 | 67.61 | 12.50 |
| 1400 | 55.25113 | 56.93093 | 0.16082 | 12.891723 | 22.20 | 8.80 | 92.37 | 10.33 |

# Data using for WEIGHTED PLATEAU & INVERSE ISOCHRON calculating.
